# Unpredictable Metastasis in the Head and Neck Region: A Diagnostic Immunohistochemical Challenge

**DOI:** 10.3390/diagnostics13233513

**Published:** 2023-11-23

**Authors:** Raluca-Maria Closca, Adrian Nicoara, Marina Rakitovan, Ion Cristian Mot, Flavia Baderca

**Affiliations:** 1 Department of Microscopic Morphology, University of Medicine and Pharmacy “Victor Babes”, 300041 Timisoara, Romania; raluca.moaca@umft.ro (R.-M.C.); baderca.flavia@umft.ro (F.B.); 2 Department of Pathology, Emergency City Hospital, 300254 Timisoara, Romania; 3 Oro-Maxillo-Facial Surgery Clinic, Emergency City Hospital, 300062 Timisoara, Romania; marina.rakitovan@umft.ro; 4 Discipline of Dentoalveolar Surgery, University of Medicine and Pharmacy “Victor Babes”, 300041 Timisoara, Romania; 5 Otorhinolaryngology Clinic, Emergency City Hospital, 300254 Timisoara, Romania; mot.ion@umft.ro; 6 Department IX Surgery, University of Medicine and Pharmacy “Victor Babes”, 300041 Timisoara, Romania

**Keywords:** head and neck, unusual metastases, immunohistochemistry, renal clear cell carcinoma, hepatocellular carcinoma

## Abstract

Metastatic disease is a complex and sequential process that involves the migration of tumor cells from the primary site to distant areas. This metastatic pathway is not always predictable. Therefore, this paper presents three rare cases of unusual metastases, due to their primary site: two metastases of a clear cell renal cell carcinoma, one gingival, and one nasal, as well as a mandibular metastasis of a hepatocellular carcinoma. In all cases, an incisional biopsy was performed in order to find out the diagnosis. After microscopical examination of morphological Hematoxylin and Eosin-stained slides, for all cases, immunohistochemical reactions were performed to support the primary tumor site. Two cases had a previous histopathological diagnosis of a primary tumor, while for the third case, the metastatic lesion represented the first manifestation of the neoplastic disease, with an unfavorable prognosis.

## 1. Introduction

Clear cell renal cell carcinoma is the most common subtype of renal carcinoma and usually gives way to hematogenous metastases in the brain, lung, liver, and bone via the inferior vena cava. Renal cell carcinoma metastasis in the head and neck organs is uncommon, accounting for only 6% of metastases, most commonly involving the thyroid, parotid gland, and sinuses [1,2]. If the disease is advanced, the risk of metastasis in the head and neck region is raised, being about 15%, while in localized disease, the risk is only around 1% [3]. The data show that renal cell carcinoma is the third most common infra clavicular malignancy to metastasize to the head and neck region. Most cases described in the English literature presented a history of primary renal tumor, and patients often had advanced metastatic disease [4,5].

Hepatocellular carcinoma is the most common primary hepatic tumor and the sixth most common malignancy in the world. It is considered the second most common cause of death from cancer worldwide. The improvement in intrahepatic tumor control and prolonged survival has led to an increased incidence of extrahepatic metastasis. The most frequent sites of metastasis are bones, followed by the lungs and lymph nodes. Oral bone metastasis is a soft-tissue mass, which may occur with pain, swelling, and local mass effects of osteolysis, hindering the patient’s quality of life. Radiotherapy can be used as a palliative treatment, and its efficacy in this regard has been reported in previous studies. The most common sites of bone metastasis from hepatocellular carcinoma are the spine, pelvis, ribs, and long bone. About 1% of hepatocellular carcinoma metastases show oral involvement, with the most frequently affected site being the posterior angle of the mandible. Less common metastatic sites, such as the mandible, were first reported by Dick et al. in 1957. Since then, mandibular metastases of hepatocellular carcinomas have been described in several case reports and small case series. Consequently, there have been few reports on the role of radiotherapy in these rare cases, which is the main palliative treatment for a bone metastasis from hepatocellular carcinoma [6,7,8].

The presence of metastases in the head and neck region from the primary hepatic or renal site is a multistep process and dynamically controlled by epithelial–mesenchymal transition (EMT), induced by transforming growth factor β (TGF-β) and the functions of microRNAs and long noncoding RNAs. The future is reserved for targeted therapies based on these characteristic molecular changes [9].

Head and neck metastases of hepatic and renal carcinoma are very rare and unusual with nonspecific symptomatology, which leads to a delayed diagnosis. Therefore, the prognosis of metastatic liver and renal carcinoma is usually unfavorable. Due to nonspecific symptomatology, a biopsy should be performed with histopathologic examination, and an immunohistochemical study of these lesions is required.

This study assessed unconventional head and neck metastases of clear cell renal cell carcinoma and hepatocellular carcinoma, highlighting the importance of the immunohistochemical profile in order to establish the primary site of the tumor, especially in cases with an unknown primary tumor.

## 2. Case Presentation

### 2.1. Case 1

#### 2.1.1. Clinical Findings

A 67-year-old male patient presented to the Oro-Maxillo-Facial Surgery Clinic in January 2023 with a soft-tissue mass located on the right mandibular alveolar ridge. The lesion had an irregular shape and was 1.5/2/3 cm in size. At palpation, mobility to the underlying mandibular bone structure was noted, with a discrete hemorrhagic phenomenon at the time of examination. The patient denied local sensitivity, spontaneous pain, or any disturbances. The onset of symptoms was approximately three years ago. The patient had no history of neoplastic disease.

The native computed tomography scan revealed the presence of a mandibular tissue mass, without any osteolysis, and upper-right cervical lymph node enlargement, 1.7 cm, respectively, upper-left cervical lymph node enlargement, 1 cm in diameter. Due to the high blood level of the creatinine value, intravenous contrast media could not be used.

Under local anesthesia, an incisional biopsy of the mandibular tissue mass was performed, and the harvested fragments were fixed in 4% *v*/*v* buffered formaldehyde, sent to the Service of Pathology, and processed with the usual histological technique. Four-micrometer-thick sections were cut using a semi-automated Leica RM2235 rotary microtome, displayed on SuperFrost™ microscope (St. Louis, MO, USA) slides, and stained with Hematoxylin and Eosin (HE).

#### 2.1.2. Pathological Findings

**Figure 1:**
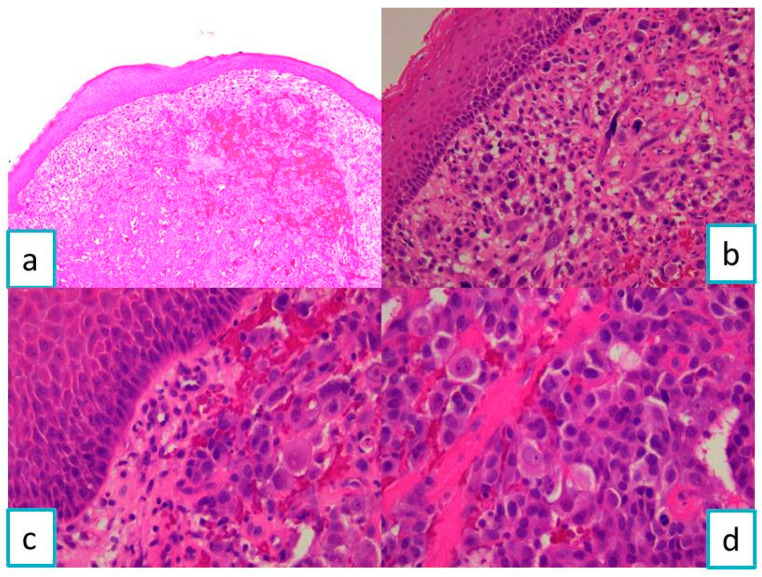
Microscopic imaging—HE-staining of gingival mucosa with tumor proliferation: (**a**) ob. 5×, (**b**) ob. 20×, and (**c**,**d**) ob. 40×.

The histopathological examination in Hematoxylin–Eosin staining revealed a malignant tumor proliferation consisting of large polyhedral cells, arranged in nests and lobules, with important cytonuclear atypia. Tumor cells had large, pale cytoplasm and enlarged oval or irregularly contoured pleomorphic and vesicular nuclei, with eosinophil macronucleoli. The tumor stroma showed fine connective septa with a delicate vascular network and extravasated erythrocytes (Figure 1).

A presumptive diagnosis of malignant tumor was made. The immunohistochemical (IHC) reactions were requested in order to confirm the phenotype of the tumor cells and to differentiate between carcinoma, achromic melanoma with epithelioid cells, leiomyosarcoma or rhabdomyosarcoma, angiosarcoma, and even a Kaposi’s sarcoma, and the site of primary malignancy.

All the data regarding the antibodies used for IHC reactions are provided in Table 1.

The tumor cells were immunohistochemically positive for CK AE1/AE3 and vimentin (Vim) and negative for S100 protein, HMB45, Melan-A, SMA, desmin, CD31, CD34, and HHV8, confirming the epithelial origin of the tumor.

The next step was to establish the cytokeratin profile of the tumor using cytokeratins 7, 20, 8/18, and 5. Due to the concomitant positivity of tumor cells for pan cytokeratin AE1/AE3 and vimentin, the antibodies panel was completed with EMA (epithelial membrane antigen), RCC (renal cell carcinoma), and CD10.

**Figure 2:**
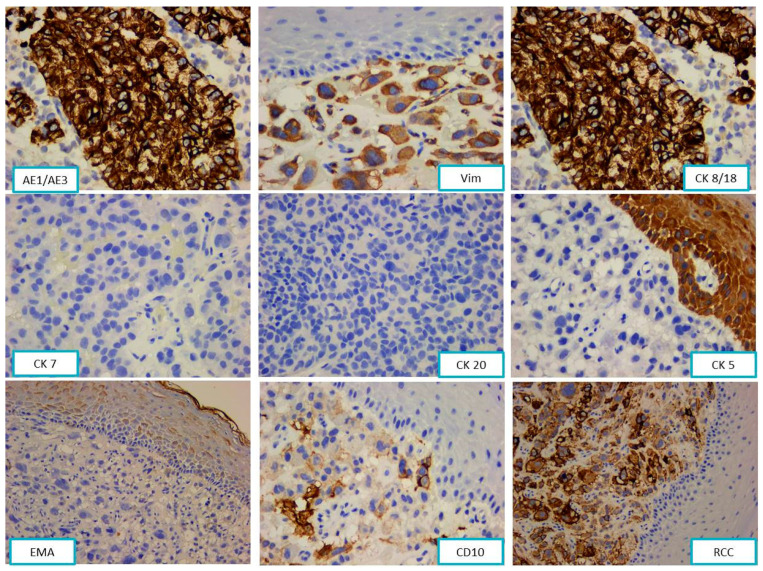
The tumor cells were immunohistochemically positive for CK AE1/AE3, vimentin, CK 8/18, CD10, and RCC and negative for CK7, CK20, CK5, and EMA.

The tumor cells’ phenotypes were CK AE1/AE3+, CK8/18+, vimentin+, RCC+, CD10+, CK7−, CK20−, CK5−, and EMA− (Figure 2).

**Figure 3:**
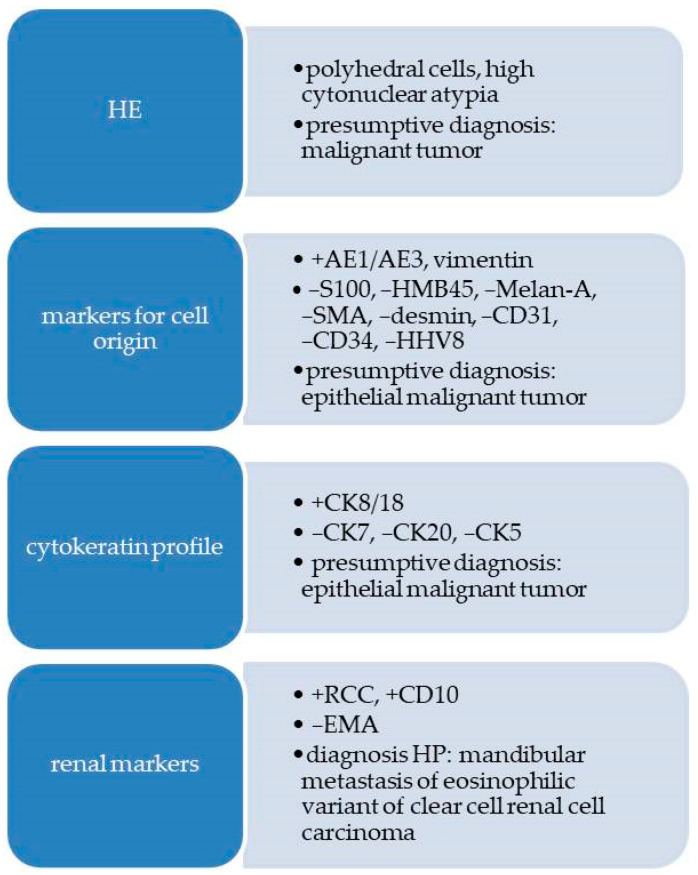
Flow diagram with morphological and immunohistochemical aspects for Case 1.

A flow diagram with the morphological and immunohistochemical reactions is presented in Figure 3.

Based on the immunohistochemical profile, the diagnosis of mandibular metastasis of the eosinophilic variant of clear cell renal cell carcinoma was established and the patient underwent oncological consultation, radiotherapy, and clinical and oncological follow-up. 

### 2.2. Case 2

#### 2.2.1. Clinical Findings

A 79-year-old female patient, with a history of clear cell renal cell carcinoma, diagnosed and surgically removed with free margins approximately 20 years ago, presented herself to the Otorhinolaryngology Department in January 2021 with a polypoid tissue mass at the level of the nasal fossa. The lesion was 1/0.9/0.7 cm in size. The lesion was painless, but slightly hemorrhagic at the exploratory examination. The patient observed the occurrence of the lesion six months prior to the presentation, with a slow-growing character.

Under local anesthesia, an excisional biopsy of the tissue mass was performed, and the harvested fragments were sent to the Service of Pathology in buffered formaldehyde and processed with the usual histological technique. Four-micrometer-thick sections were cut using a semi-automated Leica RM2235 rotary microtome, displayed on SuperFrost™ microscope slides and stained with HE. 

#### 2.2.2. Pathological Findings

**Figure 4:**
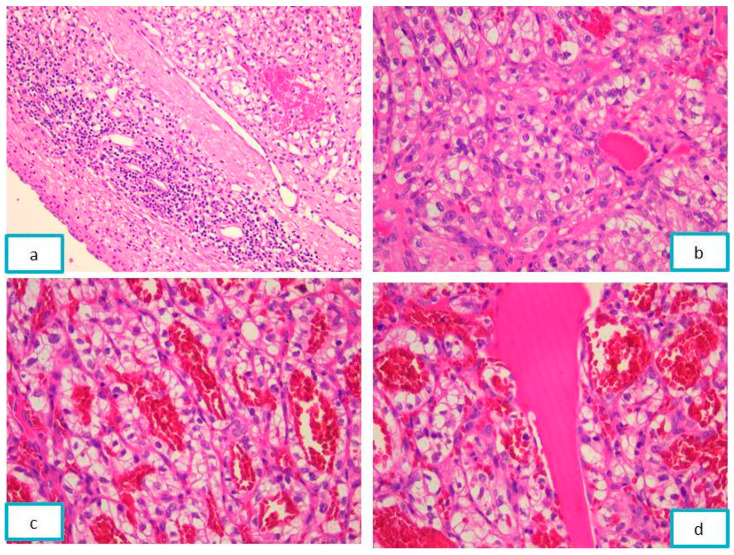
Nasal mucosa with tumor proliferation, with acinar and a nested pattern, areas of hemorrhage, and cyst formation, on the HE-stained slide: (**a**) ob. 5×, (**b**) ob. 20×, and (**c**,**d**) ob. 40×.

The histopathological examination of the Hematoxylin–Eosin-stained slides revealed a malignant tumor proliferation consisting of large polyhedral cells with compact and alveolar cellular arrangements, and a moderately clear cytoplasm, rounded nucleus, and fine granular chromatin pattern with small inconspicuous nucleoli. The malignant cells delineated a variable sized cyst filled with eosinophilic acellular material. The tumor stroma showed delicate fibrous septa, a rich, branched vascular network, and areas of fibrinoid necrosis (Figure 4). 

On HE-stained slides, the presumptive diagnosis of clear cell carcinoma was raised. Based on the personal history of the patient, the suppositional diagnosis of nasal septum metastasis from clear cell renal cell carcinoma was made.

The histopathological diagnosis was completed using IHC reactions performed in order to phenotype the tumor cells and to sustain the renal origin. All the data regarding the antibodies used for IHC reactions are reported in Table 2.

**Figure 5:**
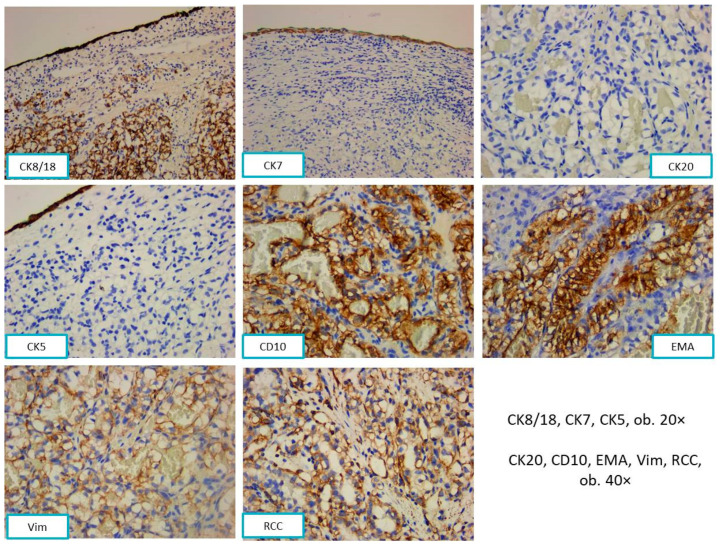
The tumor cells were positive for CK8/18, CD10, EMA, RCC, and vimentin, and negative for CK7, CK5 (with internal positive control of the nasal mucosa), and CK20.

The tumor cells showed positivity for CK8/18 and negativity for CK7, CK20, and CK5. The positive reactions for EMA, CD10, vimentin, and RCC sustained the renal origin (Figure 5). 

**Figure 6:**
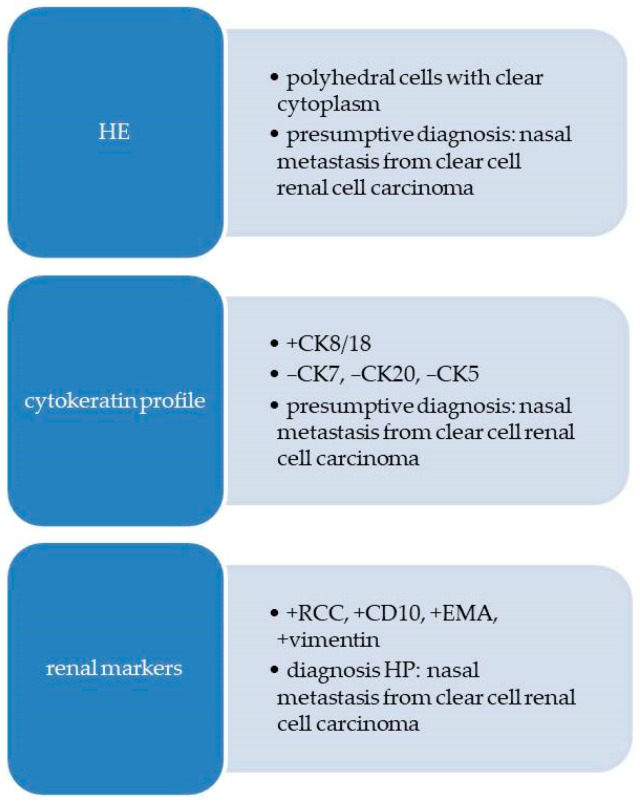
Flow diagram with morphological and immunohistochemical aspects for Case 2.

A flow diagram with the morphological aspects and immunohistochemical steps is presented in Figure 6.

Based on the immunohistochemical profile and the history of clear cell renal carcinoma, the diagnosis of nasal metastasis of a clear cell renal cell carcinoma was signed off. After the diagnosis of nasal metastasis, the patient underwent oncological consultation, radiation therapy, as well as clinical and oncological follow-up.

### 2.3. Case 3

#### 2.3.1. Clinical Findings

A 63-year-old male patient, with several comorbidities (cerebrovascular accident and high blood pressure), presented in March 2021 at the Oro-Maxillo-Facial Surgery Clinic citing the presence of an abnormal mass with a spontaneous pain response and on palpation in the right masseter region, of about 3–4 cm, imprecisely delimited, apparently fixed to the underlying mandibular bone plane, and of relatively low consistency. The patient established the appearance of the lesion as two months before the current presentation, with a continuous and rapid growth. In the past, the patient had undergone surgical removal (in 2018) and radio-chemotherapy oncological treatment (in 2019) for a liver carcinoma.

**Figure 7:**
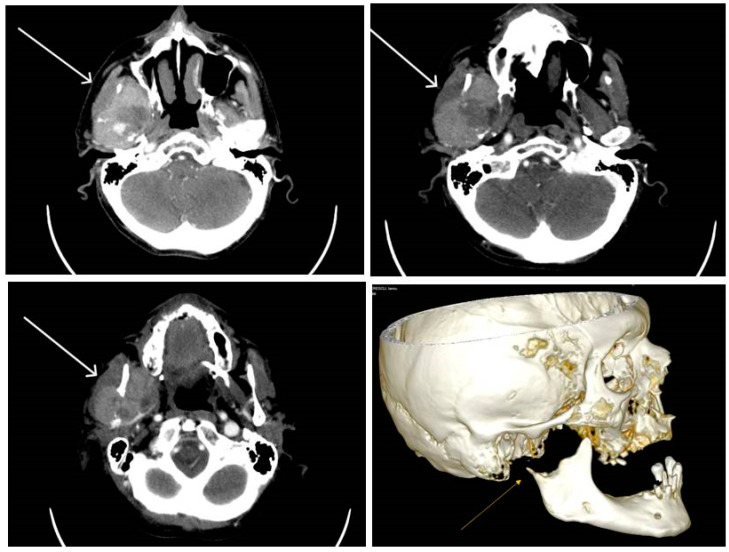
Computed tomography imaging—heterogeneous tissue mass with necrotic areas at the level of the right masticatory space (arrow) with lyses of the mandibular condyle.

The computed tomography scan with contrast media revealed the presence of a tissue mass in the masticatory space on the right side. The dimensions of the lesion were 5.5/3.6 cm in the axial plane. The lesion was moderately inhomogeneous, because of the necrotic areas, most probably with a mandibular starting point that infiltrated both the masseter and adjacent pterygoid muscle that lyses the mandibular condyle and the proximal segment of the vertical branch of the right mandible, avoiding the ipsilateral parapharyngeal space. The formation did not show a definite delimitation from the right parotid gland, instead it showed a lack of visualization of the jugular vein on the right side and no significant cervical lymph node enlargement (Figure 7).

An incisional biopsy was performed and several tumor samples were taken using an extraoral approach. The collected tissue, fixed in buffered formalin, was sent to the Service of Pathology of Timisoara’s Emergency City Hospital for the histopathological examination and processed with the usual histological technique. Four-micrometer-thick sections were cut using a semi-automated Leica RM2235 rotary microtome, displayed on SuperFrost™ microscope slides, and stained with HE.

#### 2.3.2. Pathological Findings

**Figure 8:**
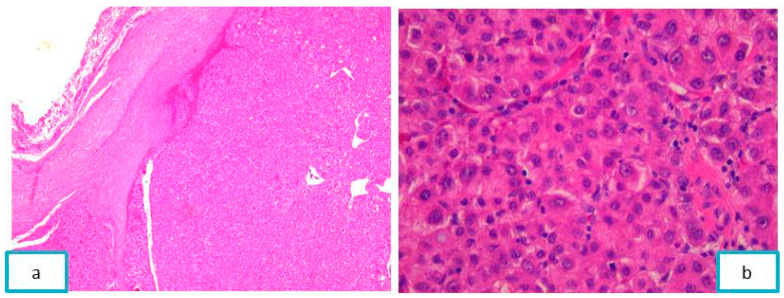
(**a**) Tumor circumscribed by a pseudo-capsule of fibrous connective tissue, ob. 5×. (**b**) Tumor cells with a trabecular pattern and sinusoidal-like spaces, HE-staining, ob. 40×.

Microscopic examination revealed a cellular proliferation with a trabecular pattern, composed of polygonal cells, with moderate cytonuclear pleomorphism and abundant granular eosinophilic cytoplasm. Some tumor cells were binucleated, with large, rounded nuclei, with inconspicuous nucleoli and coarsely chromatin. Tumor cells bordered optically empty “sinusoidal-like” spaces. The tumor was circumscribed by a pseudo-capsule of fibrous connective tissue (Figure 8).

The histopathological diagnosis was completed using immunohistochemical reactions in order to sustain the presumptive diagnosis of hepatocellular carcinoma. All the data regarding the antibodies used for IHC reactions are shown in Table 3.

**Figure 9:**
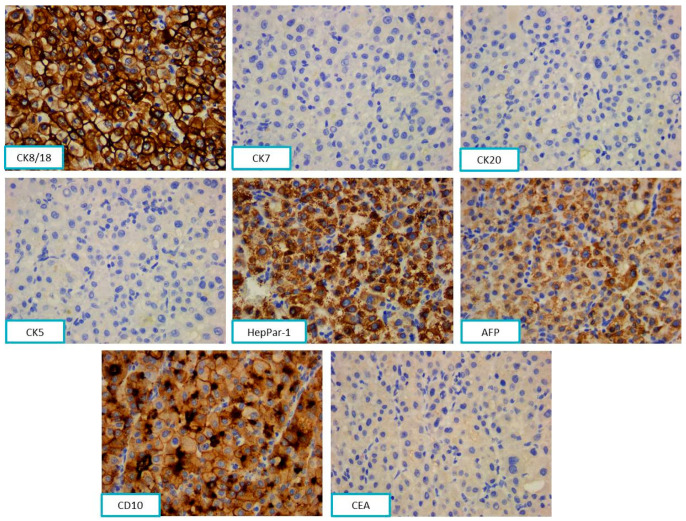
The tumor cells were positive for CK8/18, HepPar-1, AFP, and CD10 and negative for CK7, CK20, CK5, and CEA, ob. 40×.

The cytokeratin profile showed CK 8/18 positivity and CK7, CK20, and CK5 negativity. The hepatic origin was sustained by immunohistochemical positivity for HepPar-1, AFP, and CD10 and negativity for monoclonal CEA (Figure 9).

**Figure 10:**
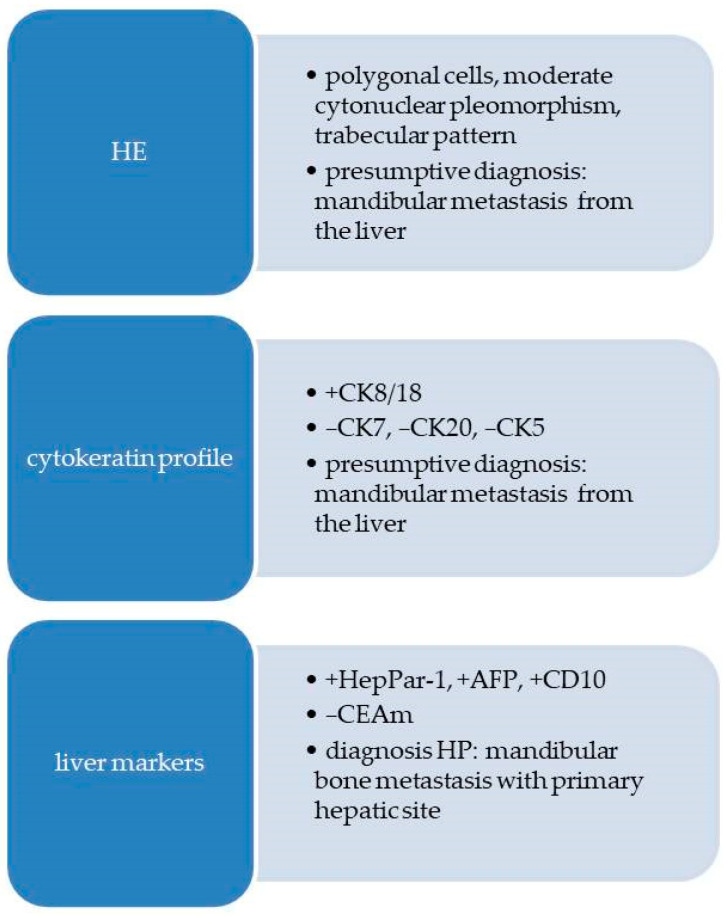
Flow diagram with morphological and immunohistochemical aspects for Case 3.

Based on the HE microscopic aspects of the tumor and the history of hepatocellular carcinoma, mandibular metastasis from the liver tumor was suspected. A flow diagram with the morphological and immunohistochemical reactions for Case 3 is presented in Figure 10.

The morphological aspects correlated with the immunohistochemical profile, associated with the patient’s history, established the final diagnosis of mandibular bone metastasis with primary hepatic site. The patient went for an oncological consultation and then radiotherapy, followed by clinical and oncological check-ups.

The main anamnestic data as well as the outcomes of the three patients presented previously can be found in Table 4.

## 3. Discussion

The presence of unusual metastases in the head and neck region is a complex process controlled by epithelial–mesenchymal transition (EMT) induced by transforming growth factor β (TGF-β) and the functions of microRNAs and long noncoding RNAs. The epithelial to mesenchymal transition is a mechanism by which neoplastic cells lose polarity, decrease adhesion, and adopt the characteristics of a mesenchymal phenotype, becoming motile and invading distant sites. Key TGF-β-induced effectors in this process are the transcriptional repressors of E-cadherin, respectively, SNAIL1, SNAIL2, ZEB1/2, and TWIST [9,10].

Researchers found that a long noncoding RNA (lncRNA) activated by TGF-β (lncRNA-ATB) induces EMT and invasion by competitively binding miR-200 family members, which promoted organ-specific metastasis by binding IL-11 mRNA. This competitive binding increases IL-11 mRNA stability and causes autocrine induction of IL-11 and subsequent activation of STAT3 signaling. These findings suggest that lncRNA-ATB, a mediator of TGF-β signaling, could predispose hepatocellular carcinoma patients to distant metastasis [9,10].

This mediator of TGF-β signaling is also correlated with metastases and promotes cell migration and invasion in renal cell carcinoma. Knockdown could inhibit cell proliferation, trigger apoptosis, reduce EMT programming, and suppress cell migration and invasion [9,11].

Thus, miRNAs and long noncoding RNA are emerging as potentially quantifiable biomarkers of cancer status and are potential targets for anti-metastatic therapies.

### 3.1. Metastases of Clear Cell Renal Carcinoma

Renal cell carcinoma is the sixth most common cancer in men and the tenth most common in women, with a higher incidence and a wide variety of environmental and genetic risk factors associated with its development [1,12]. Common symptoms include palpable abdominal mass and hematuria, but most of the patients are asymptomatic until there is disseminated metastatic disease. Renal carcinoma is an aggressive malignancy, with about 40% of patients having metastatic disease at the time of diagnosis [1]. Clear cell renal cell carcinoma is the most common microscopic subtype of renal cell carcinoma, characterized by malignant, large epithelial cells with abundant clear cytoplasm. It has a higher preference for vascular invasion than for lymphatic invasion, which leads to a higher incidence of renal vein involvement. This is associated with an increased likelihood for distant metastasis rather than regional lymphatic spread, which is the common pattern of spread for other subtypes of renal cell carcinoma [4]. Renal metastases in the head and neck areas are uncommon, accounting for 6% of metastases, most commonly involving the thyroid, parotid gland, and sinuses [1,2]. 

The presence of a sinonasal or gingival proliferation with clear cells is a diagnostic challenge for the pathologist, especially in the absence of a history of clear cell carcinoma, with differential diagnoses including squamous cell carcinoma with clear cell change, mucoepidermoid carcinoma, salivary clear cell carcinoma, epithelial–myoepithelial carcinoma, achromic melanoma, alveolar rhabdomyosarcoma, and metastatic clear cell variant of follicular thyroid carcinoma [13,14,15,16].

Sinonasal renal cell-like adenocarcinoma occurs from the small seromucous glands and has similar cytologic characteristics to clear cell renal carcinoma. In the case of clear cell tumor proliferation, a previous nephrectomy or computed tomography to assess the kidney is necessary, but the diagnosis is always supported by immunohistochemistry [17]. Sinonasal renal cell-like adenocarcinoma was described for first time by Zur et al. in 2002, with the terminology of “renal cell-like tumor”, based on its similar aspects to renal clear cell carcinoma. The World Health Organization classification of Head and Neck Tumors 4th edition included this rare tumor as a morphologically unique entity of low-grade sinonasal, non-intestinal-type adenocarcinomas [18,19].

Microscopically, renal clear cell carcinoma metastases present medium-sized cells, with compact and alveolar cellular arrangements, as well as a moderate clear cytoplasm, rounded nucleus, and fine granular chromatin pattern, with small inconspicuous nucleoli. The tumor stroma shows delicate fibrous septa and a rich, branched vascular network, while sinonasal renal cell-like adenocarcinoma is composed of mixtures of follicular to glandular structures with intervening fibrous septa, without an abundant vascular network. The neoplastic cells are uniform, cuboidal to polyhedral, with abundant clear to eosinophilic cytoplasm. There are slightly irregular to shrunken nuclei with coarse chromatin. Sinonasal renal cell-like adenocarcinoma is positive for CK7 and negative for vimentin, with CD10 of variable expression, while in the selected case, the tumors cells were CD10, CK8/18, and CK AE1/AE3 intense and diffuse positive, with a negative reaction for CK7 and CK5. EMA can also be expressed in both pathologies. Therefore, the morphological and immunohistochemical aspects should be correlated with the clinical, anamnestic, and radiologic data to define the final diagnosis [20,21].

Squamous cell carcinoma with clear cell changes microscopically consists of islands and lobules of polyhedral cells with intense eosinophilic cytoplasm and nuclei with condensed chromatin on the nuclear membrane, yielding a vesicular appearance and multiple nucleoli, marked cytonuclear pleomorphism, mitoses, unicellular keratinization, or parakeratotic pearls. The cells present an intense positive immunohistochemical reaction for CK5. 

Mucoepidermoid carcinoma is an admixture of epidermoid, squamous cells, some with mucinous differentiation and goblet cells. The intracytoplasmic mucin is present in both mucoepidermoid carcinoma and metastasis of clear cell renal cell carcinoma, but goblet curls and macro-cysts filled with extravasated mucin are not characteristic of clear cell renal cell carcinoma.

Salivary clear cell carcinoma most frequently occurs in intraoral salivary gland sites and is mainly composed of polygonal epithelioid tumor cells arranged into sheets and separated by dense hyalinized stroma.

Epithelioid melanoma, thyroid carcinoma, and epithelial–myoepithelial carcinoma were ruled out because the patients had no history of these.

It is recommended to treat sinonasal and gingival metastases of clear cell renal cell carcinoma with preoperative embolization, followed by endoscopic surgical resection and radiotherapy, which can improve the quality of life and keep the local disease under control [21,22].

### 3.2. Jaw Bone Metastasis of Hepatocellular Carcinoma

Hepatocellular carcinoma is the sixth most frequently diagnosed cancer. The management of hepatocellular carcinoma has been improved due to the enhancement of surgical and chemotherapeutic interventions. Although the life expectancy of patients with hepatocellular carcinoma has increased, distant metastasis remains a challenge in the treatment of this disease. There are two mechanisms of metastasis from the liver to the maxillofacial territory. The first mechanism involves the hepatic artery and the portal vein. When tumor tissues affect these vessels, metastatic dissemination would reach the lung first and then the maxillofacial area. It has been postulated that there may be a connection between the azygos or hemiazygos veins and the vertebral venous plexus, which creates another route for hematogenous spread. Consequently, there would be free communication between the venous systems of the neck, thorax, abdomen, and pelvis and the non-valve vertebral venous plexus, which extends from the cranial base to the coccyx. Any increase in the intra-abdominal pressure can result in an ascendant flow through the vertebral venous plexus. In such cases, hepatocellular carcinoma cells could reach the maxillofacial territory through these hematogenous routes and metastasize into the mandible [6,23,24].

In the bones of the jaw, the mandible is a frequent site for metastases (80–85%). This predisposition could be explained by the presence of a sinusoidal vascular network rich in hematopoietic tissue that favors the spread of the tumor cells. The presence of distant metastases is related to the histopathological subtype of the hepatocellular carcinoma, the degree of cellular differentiation, but also the local anatomy and tissue characteristics of the bone [25]. In the process of metastasis, dissemination of the cells from the primary site or thrombus mixed with carcinoma cells may contribute to the formation of metastatic lesions. The specific structure of the mandible, lower blood flow rates in the canal, and abrupt alteration to this can result in the permanent implantation of a tumor thrombus [26]. Additionally, Van der Waal et al. [27] found that there was an increasing risk for tumor embolus formation with low blood flow in the red bone marrow. Up to 25% of the adult mandibular marrow cavity is occupied by red bone marrow, mainly in the regions of the third molars and premolars, which become the principal targets for tumor metastasis [19].

A study conducted in 2017 by Irani on 453 patients showed a preference of jawbone metastases for the mandible. He reported a percentage of almost 74% of jawbone metastases in the mandible and showed that the primary site of jaw metastases differs by gender. Thus, in men, the primary hepatic site occupied second place after the lung, while in women it was not among the preferred sites. He reported that the time from the diagnosis of the liver tumor to the appearance of oral lesions was between 2 and 84 months. According to our data, the selected patient presented 27 months between the diagnosis of the liver tumor and the mandibular lesion [28].

In the presented case, the history of hepatocellular carcinoma and the positivity of liver cell line markers, respectively, HepPar-1, AFP, and CD10, associated with the negative reaction for CK7 and CEA, led to the diagnosis. However, there are difficult cases in which the presence of metastasis is the first manifestation of the disease, and sometimes the tumor can be poorly differentiated. In such cases, the use of cell line markers and subsequent ones specific to the tumor site are required [23,29,30].

Studies have shown increased sensitivity and specificity using a combination of markers. Hepatocellular carcinoma is positive for certain cytokeratins, particularly low-molecular-weight cytokeratins (CK8/18), and for CEA (usually with canalicular staining). HepPar-1 is an antigen reflecting hepatocytic differentiation, and it stains both normal fetal and adult livers as well as neoplastic tumors, including 80% to 100% of hepatocellular carcinomas. The staining is usually of a granular, cytoplasmic pattern. Some researchers have reported decreased staining with more poorly differentiated tumors. AFP is an oncofetal glycoprotein that is positive in approximately 70% of hepatocellular carcinoma cases. However, AFP has limitations in its sensitivity and specificity for hepatocellular carcinoma. Therefore, its use should be correlated with HepPar-1 and CD10 and the cytokeratin immunohistochemical profile [31].

The prognosis of patients with hepatocellular carcinoma metastases is poor, and the radical resection of metastases has been controversial. Palliative surgery could be performed in patients who report spontaneous bleeding, severe pain, and dysphagia caused by large tumor sizes, while palliative treatment such as radiotherapy, chemotherapy, and immunotherapy are used to relieve the pain and prolong the patient’s life [32,33].

Metastases of primary renal and hepatic origin are rare in the head and neck region. Most of the manuscripts are case reports or case series that include only a few cases, and studies that include more patients are meta-analyses. The Table 5 shows the main case series and reviews in the literature over the last decade.

## 4. Conclusions

Rarely, hepatocellular carcinoma and renal cell carcinoma metastasize to the oral cavity, and such cases have a poor prognosis due to the delay in the diagnosis. Bearing in mind the possibility of unpredictable metastases from unusual primary sites, such as the kidneys and liver, in the head and neck region, even if there is no history of these malignancies, will help pathologists to choose the antibodies panel and to include targeted markers for these primary sites. Publishing all these rare metastatic cases will help physicians to be aware of the possibility of the metastatic disease, even if the primary tumor is not known.

## Figures and Tables

**Table 1:** Data related to the antibodies used for immunohistochemical reactions in order to establish a correct diagnosis of the lesion noted in Case 1.

Antibody	Substrate	Clone	Dilution
CK AE1/AE3 ^1^	Mouse, Monoclonal	AE1/AE3	1:100
EMA ^2^	Mouse, Monoclonal	GP1.4	1:300
CK8/18 ^3^	Mouse, Monoclonal	5D3	1:100
CK5 ^4^	Mouse, Monoclonal	XM26	1:100
CK7 ^5^	Mouse, Monoclonal	307M-94	1:100
CK20 ^6^	Mouse, Monoclonal	L26	1:150
CD10 ^8^	Mouse, Monoclonal	56C6	RTU ^7^
S100 protein	Rabbit, Polyclonal	EP32	1:100
HMB45 ^9^	Mouse, Monoclonal	HMB45	1:60
Melan-A	Mouse, Monoclonal	A103	1:50
CD34 ^10^	Mouse, Monoclonal	QBEnd/10	RTU
CD31 ^11^	Mouse, Monoclonal	1A10	1:75
SMA ^12^	Mouse, Monoclonal	asn-1	1:50
Desmin	Mouse, Monoclonal	DE-R-11	1:75
Vimentin	Mouse, Monoclonal	V9	1:800
HHV8 ^13^	Mouse, Monoclonal	13B10	RTU
RCC ^14^	Mouse, Monoclonal	66.4.C2	RTU

^1^ CK AE1/AE3 (pan cytokeratin AE1/AE3); ^2^ EMA (epithelial membrane antigen); ^3^ CK8/18 (cytokeratin 8/18); ^4^ CK5 (cytokeratin 5); ^5^ CK7 (cytokeratin 7); ^6^ CK20 (cytokeratin 20); ^7^ RTU (ready-to-use); ^8^ CD10 (cluster of differentiation 10); ^9^ HMB45 (Human Melanoma Black); ^10^ CD34 (cluster of differentiation 34); ^11^ CD31 (cluster of differentiation 31); ^12^ SMA (smooth-muscle actin); ^13^ HHV8 (human herpes virus 8); ^14^ RCC (renal cell carcinoma).

**Table 2:** Data related to the antibodies used for immunohistochemical reactions in order to phenotype the tumor cells described in Case 2.

Antibody	Substrate	Clone	Dilution
EMA	Mouse, Monoclonal	GP1.4	1:300
CK8/18	Mouse, Monoclonal	5D3	1:100
CK5	Mouse, Monoclonal	XM26	1:100
CK7	Mouse, Monoclonal	307M-94	1:100
CK20	Mouse, Monoclonal	L26	1:150
CD10	Mouse, Monoclonal	56C6	RTU
Vimentin	Mouse, Monoclonal	V9	1:800
RCC	Mouse, Monoclonal	66.4.C2	RTU

**Table 3:** Data related to the antibodies used for immunohistochemical reactions in Case 3.

Antibody	Substrate	Clone	Dilution
CK8/18	Mouse, Monoclonal	5D3	1:100
CK5	Mouse, Monoclonal	XM26	1:100
CK7	Mouse, Monoclonal	307M-94	1:100
CK20	Mouse, Monoclonal	L26	1:150
CEA ^1^	Mouse, Monoclonal	II-7	RTU
CD10	Mouse, Monoclonal	56C6	RTU
AFP ^2^	Mouse, Monoclonal	C3	1:100
HepPar-1 ^3^	Mouse, Monoclonal	OCH1ES	RTU

^1^ CEA (carcinoembryonic antigen); ^2^ AFP (alpha fetoprotein); ^3^ HepPar-1 (Hepatocyte Paraffin 1).

**Table 4:** Anamnestic data and case history.

Case	Primary Neoplasm	Date	Treatment	Oral Lesion	Date	Treatment
1	-	-	-	Alveolar ridge	January 2023	Radiotherapy and oncological follow-up
2	Kidney	May 2003	Resection and oncological follow-up	Nasal fossa	January 2021	Radiotherapy and oncological follow-up
3	Liver	December 2018	Resection, radiotherapy, and oncological follow-up	Mandible	March 2021	Radiotherapy and oncological follow-up

**Table 5:** Case series and reviews of the literature.

Author	Year of Publication	Journal	Number of Patients
Irani S. [28]	2017	*J. Int. Soc. Prev. Community Dent.*	453
Lieder A. [2]	2017	*Int. Braz. J. Urol.*	22
Nawale K. [25]	2016	*J. Oral Maxillofac. Pathol.*	12
Pesis M. [7]	2014	*J. Craniomaxillofac. Surg.*	41
Zhao W. [17]	2014	*Int. J. Clin. Exp.*	6

## Data Availability

The data that support the findings of this study are available from the corresponding author upon reasonable request.

## References

[1] Lenkeit C., Bank J., Shirazi M. (2020). Renal Cell Carcinoma in the Head and Neck: Case Presentation of a Patient with a Rare Metastatic Pattern. Cureus.

[2] Lieder A., Guenzel T., Lebentrau S., Schneider C., Franzen A. (2017). Diagnostic relevance of metastatic renal cell carcinoma in the head and neck: An evaluation of 22 cases in 671 patients. Int. Braz. J. Urol..

[3] Remenschneider A.K., Sadow P.M., Lin D.T., Gray S.T. (2013). Metastatic Renal Cell Carcinoma to the Sinonasal Cavity: A Case Series. J. Neurol. Surg. Rep..

[4] Ali R.A., Mohamed K.E. (2016). Metastatic Clear Cell Renal Cell Carcinoma Presenting with a Gingival Metastasis. Clin. Pract..

[5] Morita Y., Kashima K., Suzuki M., Kinosada H., Teramoto A., Matsumiya Y., Uzawa N. (2021). Differential Diagnosis between Oral Metastasis of Renal Cell Carcinoma and Salivary Gland Cancer. Diagnostics.

[6] Yu S., Estess A., Harris W., Dillon J. (2012). A rare occurrence of hepatocellular carcinoma metastasis to the mandible: Report of a case and review of the literature. J. Oral. Maxillofac. Surg..

[7] Pesis M., Taicher S., Greenberg G., Hirshberg A. (2014). Metastasis to the jaws as a first manifestation of hepatocellular carcinoma: Report of a case and analysis of 41 cases. J. Craniomaxillofac. Surg..

[8] Dick A., Mead S.G., Mensh M., Schatten W.E. (1957). Primary hepatoma with metastasis to the mandible. Am. J. Surg..

[9] Exposito-Villen A., Aranega E.A., Franco D. (2018). Functional role of non-coding RNAs during epithelial-To-mesenchymal transition. Non-Coding RNA.

[10] Hao Y., Baker D., Ten Dijke P. (2019). TGF-β-Mediated Epithelial-Mesenchymal Transition and Cancer Metastasis. Int. J. Mol. Sci..

[11] Xiong J., Liu Y., Jiang L., Zeng Y., Tang W. (2016). High expression of long non-coding RNA lncRNA-ATB is correlated with metastases and promotes cell migration and invasion in renal cell carcinoma. Jpn. J. Clin. Oncol..

[12] Capitanio U., Bensalah K., Bex A., Boorjian S.A., Bray F., Coleman J., Gore J.L., Sun M., Wood C., Russo P. (2019). Epidemiology of Renal Cell Carcinoma. Eur. Urol..

[13] Sarău C.A., Poenaru M., Balica N.C., Baderca F. (2017). Rare sinonasal lesions. Rom. J. Morphol. Embryol..

[14] Trandafir C.M., Tischer A.A., Horhat I.D., Balica N.C., Sitaru A.M., Guran K., Morar R., Baderca F., Jifcu E.M., Moţ I.C. (2020). Fortuitous discovery of melanomas in the ENT Department—A histopathological and immunohistochemical study. Rom. J. Morphol. Embryol..

[15] Baderca F., Vincze D., Balica N., Solovan C. (2014). Mucosal melanomas in the elderly: Challenging cases and review of the literature. Clin. Interv. Aging..

[16] Rakitovan M., Nicoara A., Closca R.M., Balica N.C., Stefanescu E.H., Baderca F. (2023). Leiomyoma with Uncommon Localization-Incisive Papilla and Palatal Fibromucosa: A Case Report. Medicina.

[17] Zhao W., Yangi L., Wang L., Zuo W., Shuanghu Yuan S., Yu J., Yu Q., Xudong Hu X., Wang S., Liu N. (2014). Primary clear cell carcinoma of nasal cavity: Report of six cases and review of literature. Int. J. Clin. Exp. Med..

[18] Zur K.B., Brandwein M., Wang B., Som P., Gordon R., Urken M.L. (2002). Primary description of a new entity, renal cell-like carcinoma of the nasal cavity: Van Meegeren in the house of Vermeer. Arch. Otolaryngol. Head. Neck Surg..

[19] El-Naggar A.K., Chan J.K.C., Grandis J.R., Takata T., Slootweg P.J. (2017). WHO Classification of Head and Neck Tumours.

[20] Chen Z., Wang Z., Shi H., Liu Q. (2017). Renal cell-like carcinoma of the nasal cavity: A case report and review of the literature. Diagn. Pathol..

[21] Morvan J.B., Veyrières J.B., Mimouni O., Cathelinaud O., Allali L., Verdalle P. (2011). Clear-cell renal carcinoma metastasis to the base of the tongue and sphenoid sinus: Two very rare atypical ENT locations. Eur. Ann. Otorhinolaryngol. Head Neck Dis..

[22] Sikka S., Sikka P., Kaur G., Shetty D.C. (2013). A review of histopathological and immunohistochemical parameters in diagnosis of metastatic renal cell carcinoma with a case of gingival metastasis. J. Cancer Res. Ther..

[23] Du C., Feng Y., Li N., Wang K., Wang S., Gao Z. (2015). Mandibular metastasis as an initial manifestation of hepatocellular carcinoma: A report of two cases. Oncol. Lett..

[24] Piccirillo M., Granata V., Albino V., Palaia R., Setola S.V., Petrillo A., Izzo F. (2013). Can hepatocellular carcinoma (HCC) produce unconventional metastases? Four cases of extrahepatic HCC. Tumori J..

[25] Nawale K.K., Vyas M., Kane S., Patil A. (2016). Metastatic tumors in the jaw bones: A retrospective clinicopathological study of 12 cases at Tertiary Cancer Center. J. Oral. Maxillofac. Pathol..

[26] Miller M.E., McCall A.A., Juillard G.F., Nadelman C.M., Wang M.B., Nabili V. (2013). Hepatocellular carcinoma metastatic to the mandible. Ear Nose Throat J..

[27] Van der Waal R.I., Buter J., van der Waal I. (2003). Oral metastases: Report of 24 cases. Br. J. Oral. Maxillofac. Surg..

[28] Irani S. (2017). Metastasis to the Jawbones: A review of 453 cases. J. Int. Soc. Prev. Community Dent..

[29] Chen D., Li Z., Song Q., Qian L., Xie B., Zhu J. (2018). Clinicopathological features and differential diagnosis of hepatocellular carcinoma in extrahepatic metastases. Medicine.

[30] Ruiz-Morales J.M., Dorantes-Heredia R., Chable-Montero F., Vazquez-Manjarrez S., Méndez-Sánchez N., Motola-Kuba D. (2014). Bone metastases as the initial presentation of hepatocellular carcinoma. Two case reports and a literature review. Ann. Hepatol..

[31] Dabbs D.J. (2019). Diagnostic Immunohistochemistry: Theranostic and Genomic Applications.

[32] Hong J.H., Lee K., Kim J., Ahn K.M. (2021). Prognosis of hepatocellular carcinoma metastasizing to the oral cavity. Maxillofac. Plast. Reconstr. Surg..

[33] Liu H., Xu Q., Lin F., Ma J. (2019). Hepatocellular carcinoma metastasis to the mandibular ramus: A case report. Int. J. Clin. Exp. Pathol..

